# A Case of Traumatic Hemothorax From an Isolated Thoracic Vertebral Fracture in an Elderly Patient on Combined Anticoagulant and Antiplatelet Therapy

**DOI:** 10.7759/cureus.58422

**Published:** 2024-04-16

**Authors:** Shinji Yuhara, Tadasu Kohno

**Affiliations:** 1 Department of Thoracic Surgery, National Center for Global Health and Medicine, Tokyo, JPN; 2 Department of Thoracic Surgery, New Tokyo Hospital, Chiba, JPN

**Keywords:** antithrombotic therapy, antiplatelet therapy, anticoagulant therapy, vertebral fracture, thoracoscopic surgery, traumatic hemothorax

## Abstract

Traumatic hemothorax is typically easy to diagnose because of the distinct onset of trauma with significant complaints such as severe chest pains. However, in elderly patients, the clinical symptoms are less clear and the frequent use of antithrombotic therapy may prolong the bleeding from a minor fracture. We report a case of traumatic hemothorax from an isolated thoracic vertebral fracture in an elderly patient on anticoagulant and antiplatelet therapy. A 91-year-old male on anticoagulant and antiplatelet therapy was admitted to our hospital with a complaint of persistent hemoptysis after a fall. A computed tomography (CT) demonstrated a worsening right hemothorax and thoracic vertebral fracture without lung or diaphragm injury, rib fracture, or contrast medium extravasation. The patient was taken to the operating room for the exploratory thoracoscopy and evacuation of the hemothorax without a preoperative diagnosis of the bleeding source. The bleeding was from the transverse laceration of the 10th thoracic vertebra exposed to the pleural space. The minor bleeding from the cancellous bone was prolonged, possibly due to the use of anticoagulant and antiplatelet therapy, which was not identified as contrast medium extravasation on chest CT before surgery. In cases of hemothorax with an unclear bleeding source, a vertebral fracture could be considered a source of bleeding even without any signs of bone dislocation or contrast medium extravasation on a CT scan.

## Introduction

Traumatic hemothorax can be fatal if surgical intervention is delayed. Generally, traumatic hemothorax presents with sudden onset of injury and notable symptoms, such as severe chest pain. However, in the elderly population, clinical signs may be less evident, and bleeding from minor fractures may persist due to the frequent use of anticoagulants or antiplatelet therapy. The well-known causes of hemothorax after blunt chest trauma are lung injuries, rib fractures, and damage to intrathoracic blood vessels [1]. In cases of traumatic hemothorax with the absence of typical radiological findings, such as rib fractures, lung injuries, or contrast medium extravasation, it may be difficult to diagnose the responsible bleeding source. This may delay the decision of surgical intervention especially when the surgical risk is high because of the patient’s frailty and comorbidities. However, even though the hemothorax from a fractured thoracic vertebra is rare, the consequence of delayed intervention can be fatal [2]. We report a case of traumatic hemothorax from an isolated thoracic vertebral fracture in an elderly patient on anticoagulant and antiplatelet therapy.

## Case presentation

**Figure 1:**
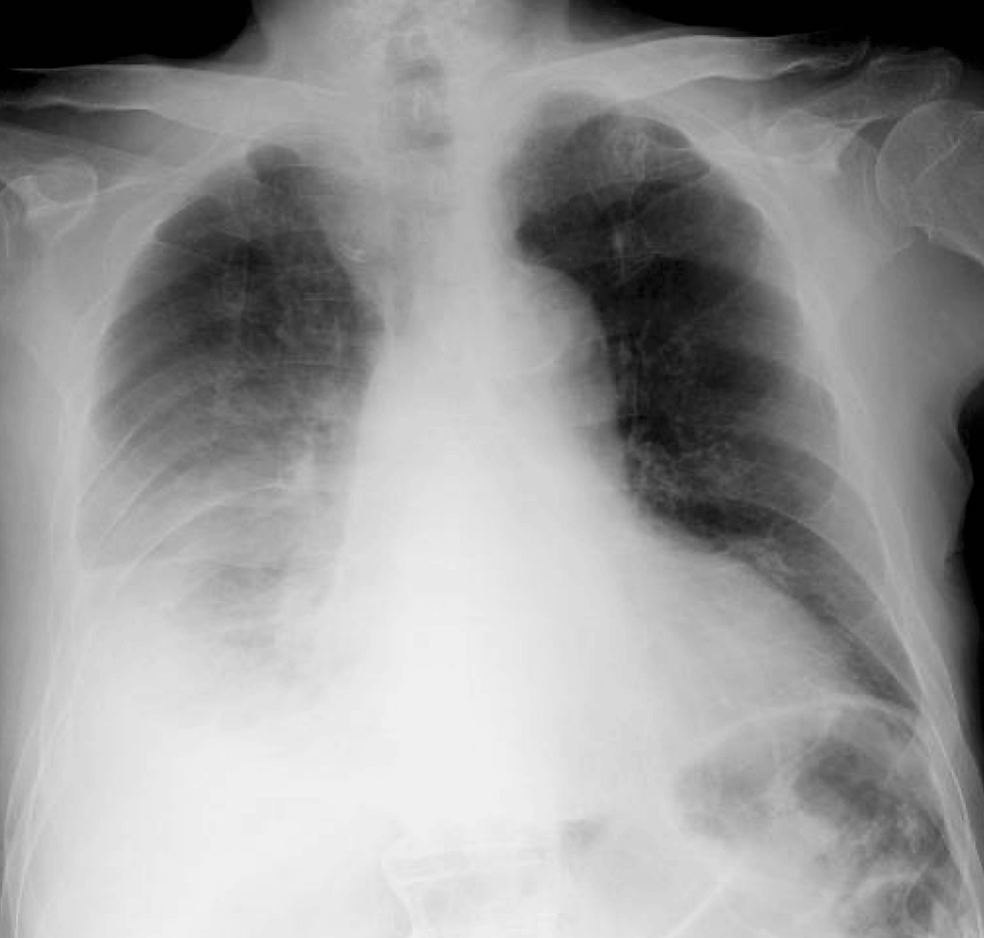
Chest X-ray on admission The chest X-ray, taken in the sitting position, showed a right massive pleural effusion.

A 91-year-old man with a medical history of myocardial infarction and cerebral infarction presented to our hospital complaining of mild chest pain and persistent hemoptysis. He had been on anticoagulant and antiplatelet therapy at the time of injury. He accidentally fell forward on the stairs and bruised his chest and abdomen one day prior. Immediately after the injury, he had mild chest pain on the right. However, several hours after the injury, he started to have hemoptysis. He presented to our hospital the next morning with persistent hemoptysis. Despite his normal vital signs, his hemoglobin had decreased from 13.4 g/dL to 10.5 g/dL since his last visit three months ago. A chest X-ray taken in the sitting position showed a right massive pleural effusion (Figure 1).

**Figure 2:**
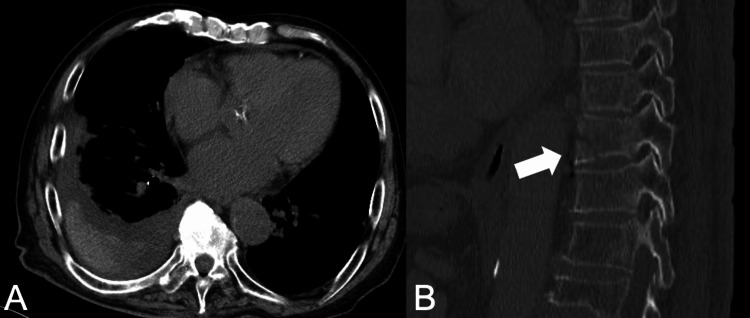
Plain CT findings on admission (A) Plain computed tomography imaging of hematoma and pleural effusion, (B) 10th thoracic vertebral fracture.

A plain computed tomography (CT) revealed right pleural effusion (Figure 2A) and a fracture of the 10th thoracic vertebra (Figure 2B) without any evidence of pneumothorax, airway injuries, lung injuries, rib fractures, or diaphragm rupture.

**Figure 3:**
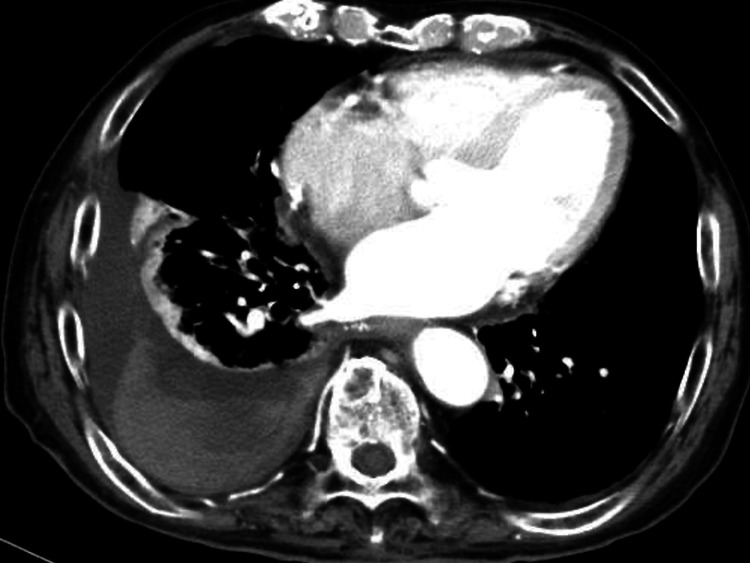
Contrast-enhanced CT findings A follow-up contrast-enhanced CT showed a worsening hematoma.

The findings of the lung and airway could be underestimated due to passive atelectasis, thus the cause of hemoptysis remained unclear. After a thorough examination and review of the CT scan by the orthopedic team, conservative management for the vertebral fracture was chosen. Given that 15 hours had passed since the injury, we also decided to manage pleural effusion conservatively without pleural drainage. Pleural fluid drainage was considered to be high risk due to his anticoagulant and antiplatelet treatment. Considering the possibility of surgery being necessary upon admission, we scheduled a follow-up blood test five hours later, which revealed a decrease in hemoglobin. Consequently, we performed a contrast-enhanced CT scan to evaluate the extent of the hematoma and identify the source of bleeding. The follow-up CT scan demonstrated a worsening hematoma without contrast medium extravasation (Figure 3).

**Figure 4:**
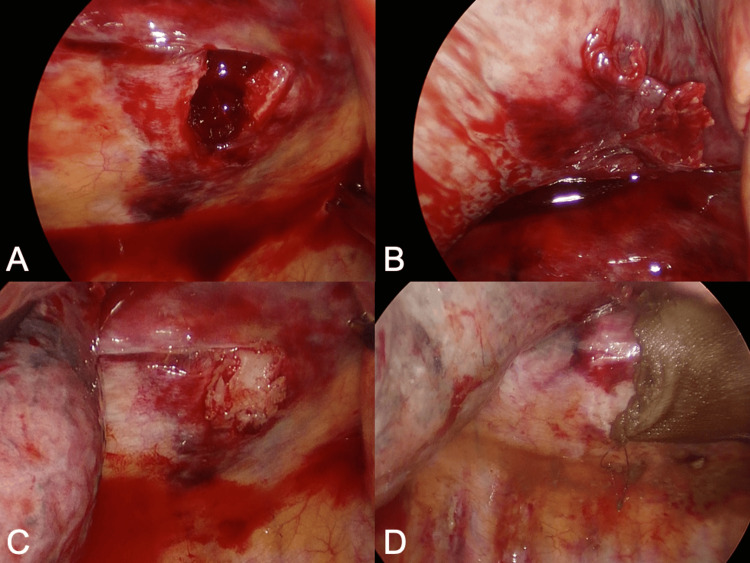
Thoracoscopic findings. (A) Bleeding from a laceration of 10th thoracic vertebra, (B) a strange soft tissue adhering to the lung, (C) hemostasis achieved by packing bone wax, (D) coverage with an oxidized cellulose sheet.

Since no interventional radiologist was available at our hospital, we opted for surgical intervention. The patient was emergently taken to the operating room for thoracoscopic exploration to identify the bleeding source. After evacuating the hematoma, we could detect ongoing bleeding from the transverse laceration of the 10th thoracic vertebra (Figure 4A). A strange soft tissue was adhered to the lung, which could be a fragment of the fractured vertebra (Figure 4B). The visceral pleura was not treated as no air leakage was detected. Hemostasis was achieved by packing bone wax into the laceration (Figure 4C) and covering it with an absorbable oxidized regenerated cellulose sheet (Figure 4D).

The surgical duration was 84 minutes, and 1400 mL of blood was lost (including the hematoma). This fracture was diagnosed as a type B3 fracture associated with diffuse idiopathic skeletal hyperostosis according to the AO classification [3]. He fell forward and bruised his chest and abdomen, which could have caused tension in the thoracic spine and led to the vertebral fracture. Due to the stability of the thoracic spine, the absence of neurological symptoms, and the patient’s comorbidities, conservative therapy was opted for. Two units of red blood cell transfusion were needed postoperatively. The chest tube was removed on the first postoperative day, and anticoagulant and antiplatelet therapy was resumed on the second postoperative day. Symptoms of hemoptysis gradually improved after surgery and completely disappeared on the fifth postoperative day. He was discharged home on the 13th postoperative day after completing in-hospital physical therapy.

## Discussion

Traumatic hemothorax is typically caused by rib fractures accompanied by lung injuries or damage to intrathoracic blood vessels. A CT scan often demonstrates rib fractures, lung parenchymal damage, or contrast medium extravasation [2,4]. Thus, identifying the cause of bleeding is not challenging. However, in our case, the CT scan did not reveal any of these typical findings but only isolated fracture of the 10th thoracic vertebra without any dislocation. As a result, we were unable to identify the bleeding source before surgery, which led to the decision to close monitoring without any immediate surgical intervention for this frail 91-year-old patient with multiple comorbidities on anticoagulant and antiplatelet therapy. However, increasing pleural effusion on CT scan and worsening anemia prompted the surgical decision. The patient was admitted to the emergency department because of hemoptysis, the cause of which was still unknown. A preoperative CT scan showed no lung or airway injury, which could have been underestimated due to passive atelectasis. It is possible that a strange soft tissue adhering to the lung was a fragment of the fractured vertebra and injured the lung. However, there is no certainty regarding the relationship between the operative findings and persistent hemoptysis. After surgery, symptoms of persistent hemoptysis improved.

There have been few reports of traumatic hemothorax due to thoracic vertebral fractures. Okamoto et al. summarized seven cases and reported that hemostasis was achieved through thoracotomy with bone wax in three cases, and spinal fusion was required in most cases. In our case, hemostasis was also achieved with bone wax via minimally invasive video-assisted surgery, and the vertebral fracture was managed conservatively.

The effect of anticoagulant therapy on the outcome of trauma patients is controversial. Sartini et al. reported a retrospective cohort study of the adverse effects of antithrombotic therapy in major trauma patients [5]. The study found that the worst survival outcomes were observed in patients with anticoagulant treatment. On the contrary, Takami et al. reported that antithrombotic treatment before injury was not associated with massive hemothorax due to thoracic vertebral fractures [6]. In this study, only AO classification type B and diffuse idiopathic skeletal hyperostosis fractures were significantly associated with massive hemothorax due to thoracic vertebral fractures, which is consistent with our case. This study included eight patients with massive hemothorax who had no injury to the great vessels or intercostal arteries and veins, pulmonary parenchymal damage, sternum injury, or rib fractures. However, as opposed to our case, a dynamic CT angiography scan was able to identify the bleeding source as the segmental artery or venous bleeding in all eight cases.

Hemothorax from thoracic spine fracture can be fatal in the elderly population. Masteller et al. reported two autopsy cases that revealed traumatic hemothorax due to bleeding from the thoracic vertebral body [7]. In both cases, a CT scan was unable to detect the bleeding source before their death until autopsy, which suggests that it may be difficult to identify hemorrhage from the laceration of the vertebral body, unlike hemorrhage from the vessels. However, the consequence of delayed diagnosis of hemothorax from the thoracic spine can be fatal in the elderly population. In our case, despite the low-energy trauma and no evidence of extravasation, surgical intervention was required for persistent hemorrhage from the laceration of the fractured 10th thoracic vertebra, which may have been caused by combined anticoagulant and antiplatelet therapy.

## Conclusions

Traumatic hemothorax can have a fatal outcome if surgical intervention is delayed. When the bleeding source is unclear in elderly patients with traumatic hemothorax who are on antithrombotic treatment, an isolated vertebral fracture should be considered as a potential cause.

## References

[1] Sirmali M, Türüt H, Topçu S (2003). A comprehensive analysis of traumatic rib fractures: morbidity, mortality and management. Eur J Cardiothorac Surg.

[2] Okamoto K, Ichinose M, Hanaoka J (2018). Traumatic hemothorax due to chance fracture requiring emergency surgical management: a report of two cases. SAGE Open Med Case Rep.

[3] Vaccaro AR, Oner C, Kepler CK (2013). AOSpine thoracolumbar spine injury classification system: fracture description, neurological status, and key modifiers. Spine (Phila Pa 1976).

[4] Ota K, Fumimoto S, Iida R (2018). Massive hemothorax due to two bleeding sources with minor injury mechanism: a case report. J Med Case Rep.

[5] Sartini S, Spadaro M, Cutuli O (2022). Does antithrombotic therapy affect outcomes in major trauma patients? A retrospective cohort study from a tertiary trauma centre. J Clin Med.

[6] Takami M, Iwasaki Y, Okada M, Nagata K, Shibata N, Kato S, Yamada H (2022). Incidence and predictive factors of massive hemothorax due to thoracic vertebral fractures. Spine Surg Relat Res.

[7] Masteller MA, Chauhan A, Musunuru H, Walsh MM, Boyer B, Prahlow JA (2012). Haemothorax and thoracic spine fractures in the elderly. Case Rep Radiol.

